# Effect of Ozone on Nonwoven Polylactide/Natural Rubber Fibers

**DOI:** 10.3390/polym17152102

**Published:** 2025-07-31

**Authors:** Yulia V. Tertyshnaya, Svetlana G. Karpova, Maria V. Podzorova

**Affiliations:** 1 Department of Biological and Chemical Physics of Polymers, Emanuel Institute of Biochemical Physics, Russian Academy of Sciences, 4 Kosygina Str., 119334 Moscow, Russia; 2 Department of Chemistry of Innovative Materials and Technologies, Plekhanov Russian University of Economics, 36 Stremyanniy, 117997 Moscow, Russia

**Keywords:** ozone treatment, polylactide, natural rubber, nonwoven fibers, polymer degradation, oxidation, diffraction patterns, FTIR-ATR spectra

## Abstract

Ozone is a powerful destructive agent in the oxidative process of polymer composites. The destructive ability of ozone depends primarily on its concentration, duration of exposure, the type of polymer, and its matrix structure. In this work, nonwoven PLA/NR fibers with natural rubber contents of 5, 10, and 15 wt.% were obtained, which were then subjected to ozone oxidation for 800 min. The effect of ozone treatment was estimated using various methods of physicochemical analysis. The visual effect was manifested in the form of a change in the color of PLA/NR fibers. The method of differential scanning calorimetry revealed a change in the thermophysical characteristics. The glass transition and cold crystallization temperatures of polylactide shifted toward lower temperatures, and the degree of crystallinity increased. It was found that in PLA/NR fiber samples, the degradation process predominates over the crosslinking process, as an increase in the melt flow rate by 1.5–1.6 times and a decrease in the correlation time determined by the electron paramagnetic resonance method were observed. The IR Fourier method recorded a change in the chemical structure during ozone oxidation. The intensity of the ether bond bands changed, and new bands appeared at 1640 and 1537 cm^−1^, which corresponded to the formation of –C=C– bonds.

## 1. Introduction

**Figure 1:**
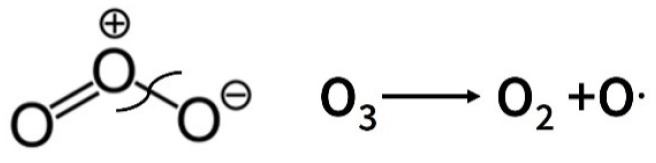
Scheme of the destruction of the ozone molecule and the formation of a radical.

The process of disintegration of materials has been studied since the middle of the last century, but even today, with the advent of new materials and new knowledge, the study of the processes of cracking and destruction of polymers is relevant [1,2,3]. Destruction can be caused by both internal and externally applied stress, and the environment that promotes destruction can be gaseous, liquid, or solid [4,5,6]. The process begins on the surface, and then cracks penetrate the volume of the polymer, and the material is destroyed. Environmental factors or directly aggressive agents play a huge role here. The influence of aggressive factors on polymers and polymer composite materials has been studied for a long time, but there is still little understanding and discussion of the effects of ozone on polymer fiber composites [7,8]. It is known that ozone is a very powerful oxidizer; it causes oxidative destruction of polymer materials, as a result of which their operational properties deteriorate [9,10]. The ozone molecule is activated by the formation of an oxygen molecule and a radical according to the scheme in Figure 1.

**Figure 2:**
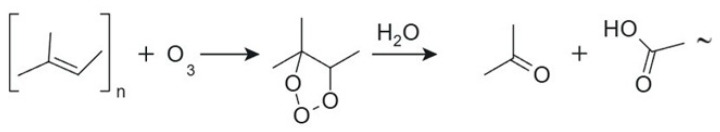
Scheme of interaction of natural rubber with ozone.

Ozone is a ground-level pollutant generated by anthropogenic activities such as combustion and the release of volatile organic compounds into the environment. Ground-level ozone concentrations have increased from <10 ppb in the pre-industrial period to 50 ppb today [11]. However, ozone can also play a positive role; for example, it is used for disinfection and sterilization in medicine or for the formation of functional groups in the modification of the surface of polymers [12,13,14,15]. Ozone is a more active oxidizer compared to oxygen. Ozone reacts with saturated C–C bonds of polymers via a free radical mechanism to form peroxide and OH radicals. Ozone reacts readily with double bonds of polyolefins, adding to them and forming cyclic structures. These structures can be reconstructed with the formation of epoxy groups and disintegrate at the site of the former double bond. The reaction rate of ozone with a saturated bond is lower than with a C=C bond [16]. It is known that the destruction of polymers with unsaturated bonds occurs according to a random law as a result of ozone attacking the double bond and the subsequent disintegration of ozonides [17], as in the case of natural rubber (Figure 2).

To understand the mechanism of aging of natural rubber (NR) under the influence of ozone, the authors selected squalene, a low-molecular-weight substance with a similar structure [18]. The ozone aging process was modeled, and changes in the molecular structure of NR were analyzed. The results showed that during the aging process, free radicals •H, •O_2_^−^, and •C=C are formed, and the NR structure is destroyed, which ultimately leads to deterioration of mechanical properties.

The ozone aging process of polylactide was also studied. The effect of ozone exposure on thermal and structural properties of polylactide-based composites [19]. Polylactide and its composites with montmorillonite were exposed to ozone at a concentration of 2.5 × 10^−5^ mol/dm^3^. The results showed that the addition of filler accelerated the degradation induced by ozone. However, the addition of montmorillonite did not change the mechanism of the degradation process [19].

In this paper, the effect of ozone on the structure and properties of non-woven fibrous material based on polylactide and natural rubber was investigated. It should be noted that there are no studies on the effect of ozone on PLA/NR fibrous matrices; therefore, in order to clarify the aging process of PLA/NR fibrous materials under the influence of ozone, a corresponding experiment was conducted, and a change in the structural characteristics was investigated. As mentioned earlier, there are studies on the effects of ozone on pure PLA and NR [2,10,20]. Treib et al. analyzed NR mixtures without and with antiozonants, p-phenylenediamine and paraffin wax, during ozone aging in the interval from 4 to 111 h. It was found that during the aging process, both antiozonants slowed down the process of NR degradation [10]. The susceptibility of PLA film samples and PLA composites to ozone was investigated in the works [2,20]. In both studies, changes in the thermophysical characteristics of PLA were observed, in particular an increase in the degree of crystallinity over the course of ozone treatment.

This is understandable because polylactide (PLA) is one of the most investigated polymers obtained from renewable natural sources [21,22,23]. Lactic acid is isolated during the fermentation of waste plant materials: beets, corn, and cereals. Then, polylactide is obtained from lactic acid by polymerization. PLA has biocompatibility and the ability to biodegrade, due to which it is used for drug delivery systems, implants, as well as for the manufacture of packaging in the food and cosmetic industries [24,25,26].

Natural rubber is a polymer of plant origin, obtained from the juice of rubber plants [27]. NR exhibits a unique combination of biodegradability, toughness, and biocompatibility. Various rubbers are used as a second component to improve the elasticity of rigid-chain polymers [28,29,30]. In this work, natural rubber was used to ensure that the resulting PLA/NR fiber composites with improved elasticity remain biodegradable and environmentally friendly. The main component of NR is 1,4-polyisoprene, which contains a large number of –C=C– double bonds and is therefore susceptible to oxidation by ozone.

The aim of this research was to explore the ozone aging of nonwoven PLA/NR fibers with different natural rubber contents. The thermophysical properties and crystal structure were studied using differential scanning calorimetry and X-ray diffraction analysis. The macromolecular mobility of PLA/NR fibers was determined by EPR. Optical microscopy and FTIR spectroscopy were used to observe morphology and structural changes, respectively. The flow rheology was evaluated by determining the melt flow rate. The combination of methods allowed us to draw conclusions about the changes in the structure and properties of non-woven PLA/NR fibers in the process of ozone oxidation, as well as about the role of NR in the degradation of composites.

## 2. Materials and Methods

### 2.1. Sample Preparation

Poly(lactic acid) PLA, 4032D, in the form of pellets with a molecular weight (M_w_) of 1.7 × 10^5^ g/mol and about 2% of D-lactide, was purchased from Nature Works (Minnetonka, MN, USA) and used as received. Natural rubber (NR), SVR-3L with Mooney viscosity 50 ± 5 (100 °C), amino acid and protein content (2–3 wt.%), poly(cis-1,4-isoprene) content (91–96 wt.%), and resins (2–3 wt.%) were supplied by Vietnam Rubber Group (Ho Chi Minh City, Vietnam).

The PLA/NR solutions for electrospinning were prepared by dissolving polymers in the right ratio (100:0, 95:5, 90:10, and 85:15) in 100 mL of chloroform. The solutions were thoroughly mixed and heated at 60 °C for about 4–5 min. For electrospinning, a vertically installed syringe with a needle with an internal diameter of 0.7 mm was used. The consumption of the solution was (9−11) × 10^−5^ g/s. The electrospinning process was carried out at room temperature (20 ± 2 °C) with a voltage of 18.5–19.5 kV. The non-woven fibers were placed on the collector. The distance between the collector and the needle was about 18 cm.

### 2.2. Differential Scanning Calorimetry (DSC)

To obtain melting thermograms, DSC analysis of fiber samples was performed on weighed samples of approximately 5.0–5.4 mg, using a DSC 204 F1 equipment (Netzsch, Selb, Germany). The non-woven fibers were heated from room temperature to 200 °C at a rate of 10 °C/min in a nitrogen atmosphere. Calibration was carried out using Indium with a melting point of 156.6 °C. The crystallinity degree of PLA (χ_c_) was determined from the first heating cycle according to Equation (1):

(1) $$\chi_{c}\left(\%\right)=\frac{{\Delta H}_{m}}{{\Delta H}_{m}^{0}\times \varphi_{PLA}}\times 100\%$$

where Δ*H*_m_ is the enthalpy of melting during heating, ${\Delta H}_{m}^{0}$ is the enthalpy assuming 100% crystalline PLA homopolymer, 93.1 J/g [31], and $\varphi_{PLA}$ is the PLA content in the composite.

### 2.3. Electron Paramagnetic Resonance

**Figure 3:**
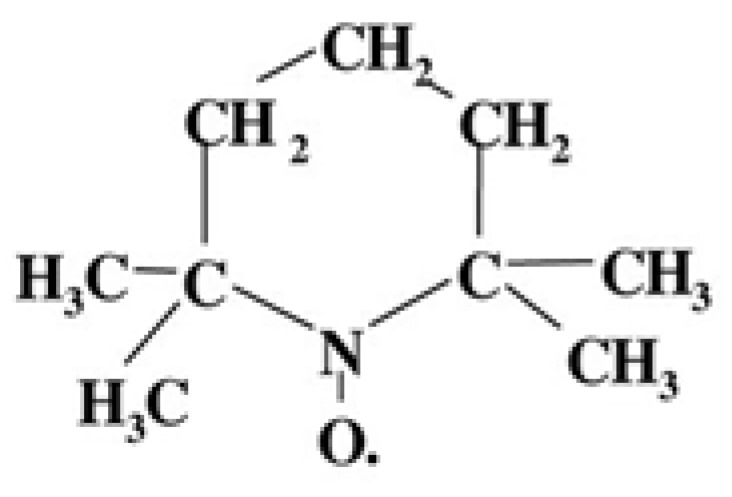
Structure of nitroxyl radical.

The macromolecular mobility was studied using electron paramagnetic resonance (EPR) by the spin probe method on an EPR-V device (Moscow, Russia). The stable nitroxide 2,2,6,6-tetramethylpiperidine-1-oxyl was applied as a probe (Figure 3).

A nitroxyl radical with a concentration of 10^−3^ M was introduced from vapors at a temperature above the glass transition temperature of polylactide (70 °C). The EPR spectra were recorded in the absence of saturation, which was checked according to the dependence of the signal intensity on the microwave field power. The obtained EPR spectra were used to calculate the probe correlation time (τ_c_) according to the following Equation (2) [32]:

(2) $$\tau_{c}\;=\;6.65\;\times \;10^{-10}\;\left(\sqrt{\frac{I_{+}}{I_{-}}}-1\right)\times ∆H_{+}$$

where ΔH_+_ is the width of the low-field component of the spectrum, and I_+_/I_−_ is the intensity ratio of low- to high-field components, respectively.

### 2.4. Morphology of the Sample

The surface microstructure of nonwoven PLA and PLA/NR fibers was characterized using an Axio Imager Z2m optical microscope (Carl Zeiss, Oberkochen, Germany). Micrographs were obtained in transmitted light. The average diameter of the fibers was determined, and the bulk density of nonwoven PLA and PLA/NR fibers was calculated according to ISO 9073-2:1995 [33].

### 2.5. FTIR Spectroscopy

The FTIR-ATR (attenuated total reflection) spectra were obtained on a Bruker Lumos IR Fourier spectrometer with 48 scans (Bruker Corp., Bremen, Germany) at room temperature in the 4000–500 cm^−1^ region. The absorption bands corresponding to vibrations of functional groups of 2995, 2945, 1750 (>C=O group), 1640, 1537, and 1300–1000 cm^−1^ were used in this work. Explanations concerning the characteristic bands are given in the text.

### 2.6. Ozone Aging

The ozone aging of fibrous samples was carried out at atmospheric pressure and a temperature of T = 21 ± 2 °C in an ozone–oxygen mixture atmosphere with a partial ozone concentration of 5.0 × 10^−5^ mol/L. The aging time varied from 0 to 800 min.

### 2.7. X-Ray Diffraction

Differences in X-ray diffraction patterns were measured using a Bruker D8 Advance (Billerica, MN, USA) diffractometer with a CuKα X-ray source and a Ni-filter in the 2Theta range of 5.0–60.0° and a step size of 0.01125°, and an exposure time of 0.15–0.25 s per step. The examined PLA/NR fiber samples were placed on a flat, low-background single-crystal silicon cuvette. The X-ray scattering intensity in the diffraction patterns was presented as a function of S = 2sinθ/λ, where θ is half the scattering angle, and λ is the X-ray wavelength.

### 2.8. Melt Flow Rate Measurement

Melt flow rate (MFR) is a parameter that allows for the evaluation of the rheological properties of polymeric materials according to GOST 11645-2021 (ISO 1133-1:2011) [34]. To determine the MFR value of nonwoven PLA/NR samples, the IIRT device (Impuls, Ivanovo, Russia) was used, the operation of which is based on the principle of a capillary viscometer. At least five samples of each composition were tested, and the average values were calculated.

### 2.9. Statistical Processing

Statistica 8.0 (Dell Software Inc., Round Rock, TX, USA) and Microsoft Excel 2010 programs were used in this work. Statistical processing consisted of calculating the arithmetic mean and its standard error. The significant differences were evaluated using Student’s *t*-test (*p* < 0.05)

## 3. Results and Discussion

### 3.1. DSC Analysis

**Figure 4:**
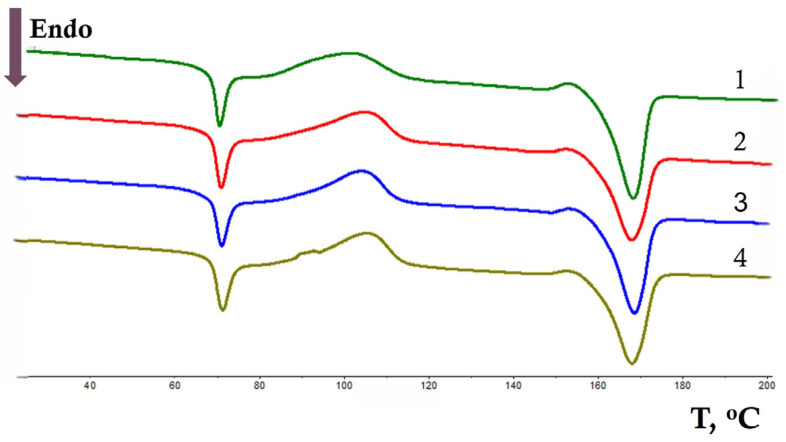
DSC thermograms of initial PLA/NR fibers with different NR content, wt.%: 0 (1), 5 (2), 10 (3), and 15 (4).

**Figure 5:**
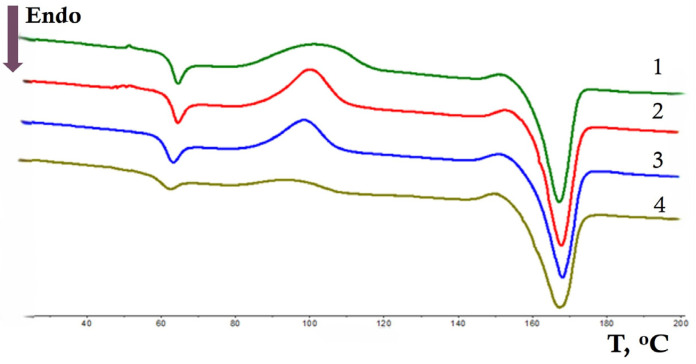
DSC thermograms of PLA/NR fibers with different NR content, wt.%: 0 (1), 5 (2), 10 (3), and 15 (4) after 800 min ozone aging.

The influence of aggressive factors always affects the structure and various properties of polymers and polymer composites. Thermophysical characteristics should also change during the oxidation process. DSC curves of the initial samples and after the action of ozone for 800 min are shown in Figure 4 and Figure 5. All thermograms contain three peaks that correspond to the glass transition temperature (T_g_), cold crystallization temperature (T_cc_), and melting temperature (T_m_) of PLA, respectively. In the obtained PLA/NR fiber samples with the addition of NR, the glass transition T_g_ increases by 1–2 °C. The change in the T_g_ of the polylactide may be a consequence of a change in the supramolecular structure, and the influence of differences in the thermal expansion coefficients of the mixed components is not excluded [35]. Table 1 summarizes the thermal properties of nonwoven PLA/NR samples after 800 min of ozone aging. The cold crystallization temperature gradually increases with increasing NR content in the samples (Table 1). Compared to the T_g_ values, the change in T_cc_ is more significant and is 4 °C for the composition containing 15 wt.% NR. The increase in T_cc_ can be explained by the increase in the amorphous phase in the composites due to the addition of NR. Natural rubber is more viscous compared to PLA. Perhaps, the presence of entanglements and the PLA-NR interphase boundary affects the cold crystallization process and crystallization in general. The melting temperature of nonwoven PLA/NR composites changes insignificantly.

When treated with ozone, the appearance of the DSC curves changes (Figure 5), namely, temperature transitions are smoothed out and temperature maxima are shifted. The glass transition temperature of pure PLA and in PLA/NR composites decreases by 5–9 °C. The value of T_cc_ has a similar tendency (Table 1). During the oxidation of polylactide and natural rubber macrochains by ozone, shorter chains are formed that are mobile at lower temperatures.

The melting point of PLA remains almost unchanged for all samples. During ozone treatment, an increase in the degree of crystallinity for all investigated fibrous samples was observed. This effect is especially evident in non-woven PLA/NR composites.

In the samples containing 5% and 10% NR, the **χ**_c_ value increases by 2 times, and in the composite with the addition of 15% NR, by 2.5 times after ozone treatment for 800 min. The effect of ozone can be similar to the process of annealing polymers [36,37,38]. It can be assumed that in the range above T_g_ but below T_m_, the mobility of macrochains increases, some stresses and entanglements in the system disappear, and rearrangements occur in the polymer structure, which are called secondary crystallization or recrystallization. The result of this process is an increase in the degree of crystallinity. In the current study, ozone has an effect instead of temperature. Its effect on the fiber structure is stronger, the higher the NR content in the polylactide matrix. It can be assumed that under the experimental conditions, ozone affects the structure of amorphous NR, the macrochains of which become defective, and has a plasticizing effect on PLA. A similar effect of increasing the degree of crystallinity of the PLA in composites after ozone processing was observed in the work [19]. The authors explained this behavior by the restructuring of the formed short chains and their increased dynamics. However, without studying the crystal structure using X-ray diffraction, it is difficult to assess the crystallinity of polymer samples.

### 3.2. X-Ray Diffraction

**Figure 6:**
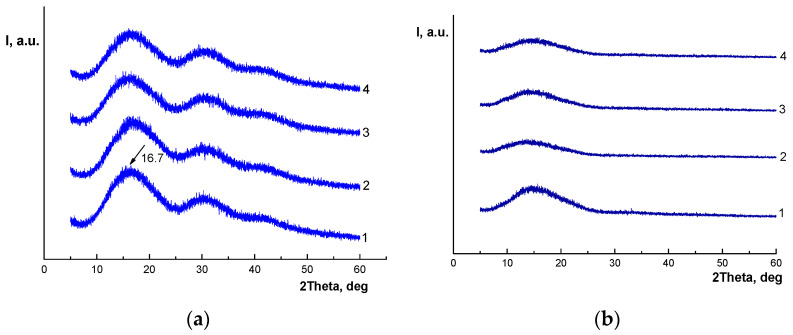
X-ray patterns of PLA/NR fibers with different NR content, wt.%: 0 (1), 5 (2), 10 (3), and 15 (4): initial (**a**) and after ozone treatment (**b**).

The XRD results complement and deepen the understanding of the crystallization process of the PLA matrix. The diffraction patterns of the initial PLA/NR fibers and after ozone aging are shown in Figure 6. It is known that PLA has intense X-ray maxima at 16.8 and 19.2 (2θ, degrees), corresponding to the α-shape of the orthorhombic cell of the crystallite [39]. According to Figure 6, no clear maxima are observed.

The structure of PLA/NR fibers is X-ray amorphous in both cases; however, after ozone treatment, the reflections became weaker, and the diffraction patterns of the initial samples and after the experiment differ significantly. These results do not indicate the perfection of PLA crystalline formations but their defectiveness in the oxidation process. The increase in the degree of crystallinity demonstrated by the DSC method may be associated with the destruction of the amorphous phase and with the participation in the crystallization process of through chains, which are “drawn” into the crystallites during ozone oxidation due to a decrease in the number of interlacings and partial breaks. Such crystalline formations are not ideal structures but are perceived by the DSC method as ordered regions that contribute to the thermal effect [40].

### 3.3. EPR (Macromolecular Mobility Study)

**Figure 7:**
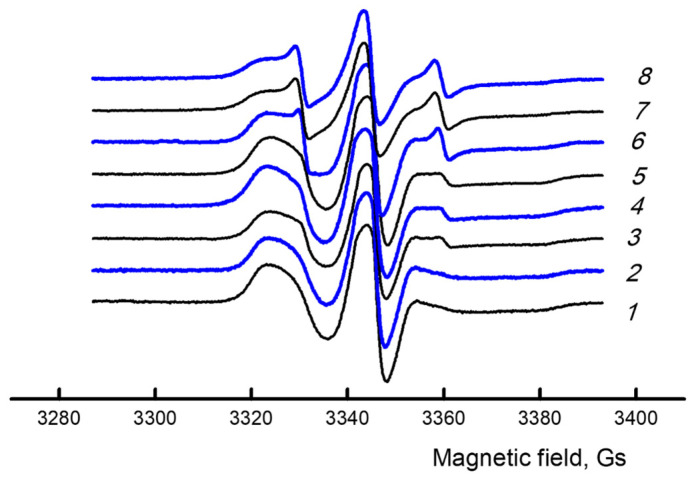
EPR spectra of PLA/NR fibers with different NR content, wt.%: initial—0 (*1*), 5 (*3*), 10 (*5*), 15 (*7*), and after ozone treatment—0 (*2*), 5 (*4*), 10 (*6*), 15 (*8*).

For a more detailed study of structural changes during ozone aging, the structural-dynamic characteristics were determined using the electron paramagnetic resonance method. Figure 7 shows the EPR spectra of the original samples and after ozonation for 800 min.

It is noticeable that the spectra of pure PLA and PLA/NR differ, especially with the addition of 10 and 15 wt.% rubber (Figure 7, spectra 5 and 7). The probe correlation time is significantly reduced in these samples, and the macromolecular mobility increases due to the increase in the total proportion of the amorphous phase (Table 2).

The effect of ozone causes two processes to occur in the polymer matrix. On the one hand, ozone oxidation leads to ruptures of defective or strained polymer macrochains, which facilitates the packing of chain “tails” into domains. On the other hand, the rigidity of the system can increase due to the formation of polar oxygen-containing groups in the side chains and intermolecular crosslinks. For example, in the work on the study of the influence of ozone on a mixture of natural rubber and chloroprene rubber, an increase in hardness was observed due to the formation of intermolecular stitches [41]. As a rule, the presence of crosslinks causes an increase in the correlation time, and the processes of degradation cause a decrease in the characteristic.

### 3.4. Morphology of Samples

**Figure 8:**
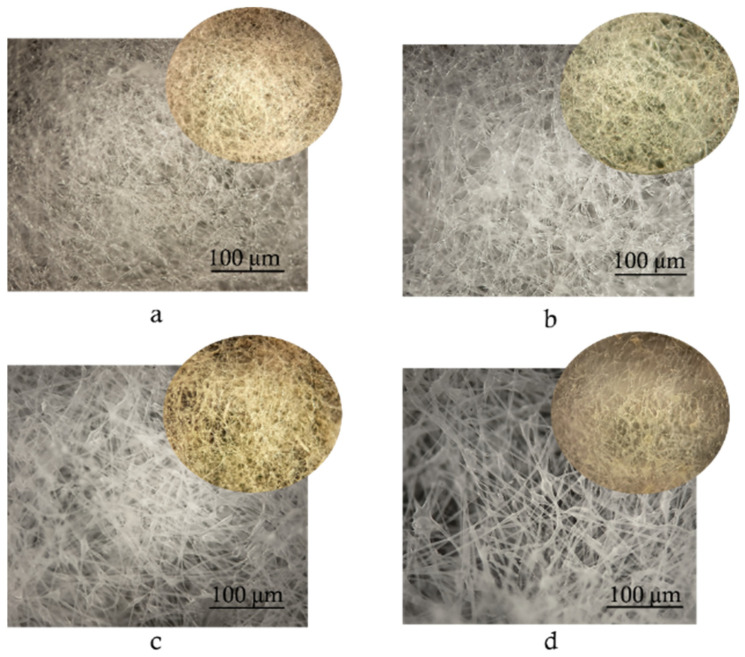
Micrographs of PLA/NR fibers with different NR content, wt.%: 0 (**a**), 5 (**b**), 10 (**c**), 15 (**d**). The insets show samples after ozone aging for 800 min.

The morphology of non-woven fibrous matrices was studied before and after ozone oxidation. The visual effect is obvious: the color of the samples changes, yellowness appears, which is one of the signs of the polymer destruction process (Figure 8). A similar color change is observed when polylactide and PLA/NR or PLA/PE composites are exposed to UV radiation [42].

The change in the color of polymeric materials is the result of chemical processes, among which the main ones can be distinguished: chemical transformations occurring in macromolecules, and the oxidation of various additives and impurities present. Also, in polymers and polymer composites, there is “a neighbor effect”. It consists of a change in the reactivity of functional groups or units under the influence of a group located nearby. Such a “neighbor” can be a more reactive component of the polymer mixture. The influence of neighbors can cause a change in the reaction rate in the polymer matrix. In the work on the study of the structure and properties of poly-3-hydroxybutyrate and ethylene propylene rubber blends, the accelerating role of rubber in the oxidation process at elevated temperatures was established [43].

The process of thermal oxidation was studied in film samples of polypropylene—polyamide 6/66 (PA6/66) blends with different component ratios. The authors observed the inhibitory effect of PA6/66 and the slowing down of the kinetics of oxygen absorption. However, in both cases, a change in the color of the polymer samples was noted during oxidation [44,45].

The obtained data on average diameters and bulk density demonstrate a tendency to decrease these values after ozone treatment compared to the initial samples of PLA/NR fibers. At this stage of the oxidation process, there is no significant drop in values, but a tendency to decrease is observed, and the value of bulk density decreases by approximately 2–4.4% depending on the composition of the fiber composites (Table 3).

### 3.5. Melt Flow Rate

**Figure 9:**
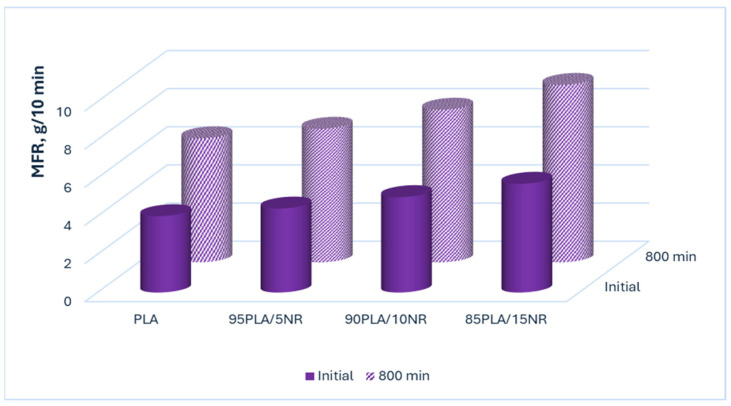
The melt flow rate (MFR) of nonwoven PLA/NR fibers: initial and after ozone treatment for 800 min.

Ozone affects not only the thermal and morphological properties of nonwoven polymer composites but also the rheological properties. The melt flow rate (MFR) was measured before and after ozone treatment for 800 min (Figure 9). In the initial fibers, the MFR value of PLA and the sample with a content of 5% NR differ little. The addition of 10 and 15% rubber slightly increases the flowability due to an increase in the content of the amorphous component. Ozone oxidation led to an increase in the MFR value of all PLA and PLA/NR nonwoven fiber samples by 1.5–1.6 times, since the degradation process affects the flowability of the fibrous material. It was previously mentioned that two parallel processes can occur in PLA/NR fiber samples: crosslinking and disintegration. When intermolecular crosslinks are formed, the viscosity of the polymer system increases, and when the destruction process occurs, the viscosity value decreases, and the MFR increases, as shown in Figure 9.

A similar result was obtained in another study. Raising the flowability of PLA/NR fiber samples was observed after their use as seed mats [46]. The MFR increased due to degradation of the polymer matrix, macrochain defects during enzymatic hydrolysis, and a decrease in viscosity.

### 3.6. FTIR-Spectroscopy

**Figure 10:**
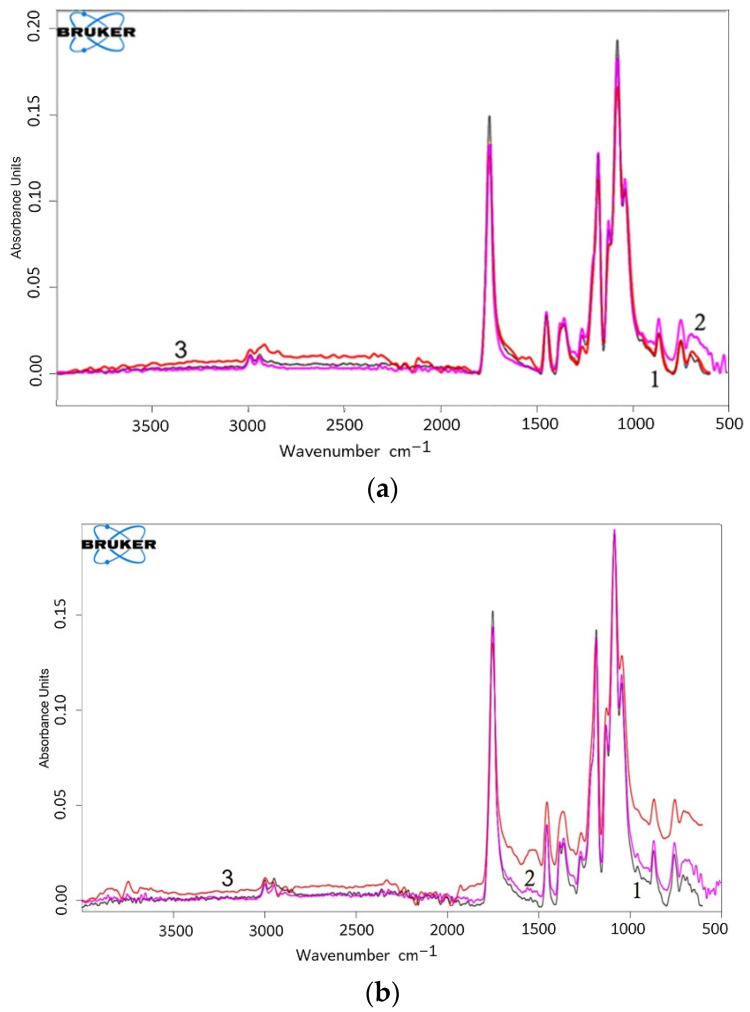
FTIR-ATR spectra of PLA (**a**) and PLA90/NR10 (**b**) samples: 1—initial (grey), 2—400 min (pink), and 3—800 min (orange) of ozone aging. Spectra of samples with 5 and 15 wt.% NR content were placed in the Supplementary file.

To record changes in the chemical structure as a result of ozonolysis, FTIR-ATR spectra were obtained before and after exposure to ozone. Figure 10 shows the IR spectrum of the original PLA and PLA/NR fibers with two distinct peaks at 2995 and 2945 cm^−1^, which are attributed to asymmetric and symmetric vibrations of the C–H stretching modes of the methyl groups (–CH_3_), as well as a group of absorption bands at 1300–1000 cm^−1^, typical of ester compounds [47]. The above bands correspond to asymmetric vibrations of C–O, C–H (–CH_3_), and symmetric vibrations of C–O and –CH_3_. Two characteristic peaks at 755 and 870 cm^−1^ are attributed to C–C vibrations in crystalline and amorphous structures of PLA, respectively [19,48]. The absorption band in the region of 1750 cm^−1^ is attributed to vibrations of C=O groups.

**Figure 11:**
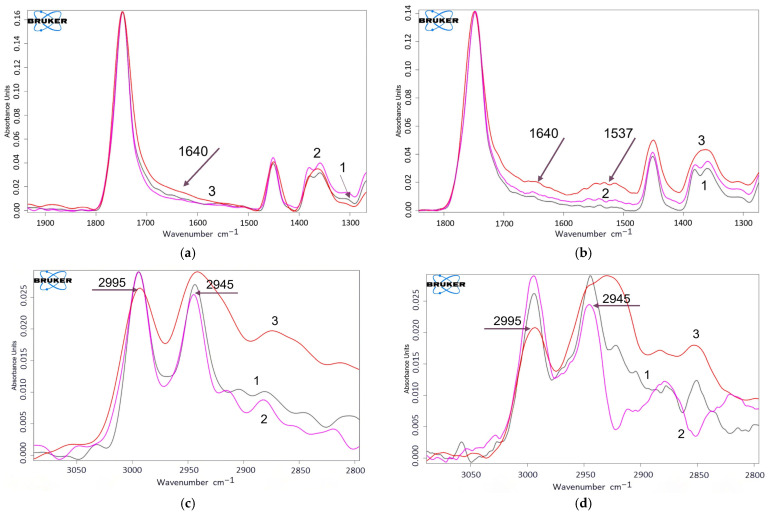
FTIR-ATR spectra of PLA (**a**,**c**) and PLA90/NR10 (**b**,**d**) samples: 1—initial (grey), 2—400 min (pink), and 3—800 min (orange) of ozone aging, 1800–1300 cm^−1^ and 3000–2800 cm^−1^.

The process of oxidative aging always causes defects and breaks in polymer macrochains and leads to the appearance of new bands in the IR spectra [48,49]. The spectra of the PLA/NR fiber samples indicate that the effect of ozone for 800 min leads to the formation of new absorption bands at 1537 and 1640 cm^−1^ (Figure 11), which correspond to the formation of C=C bonds and α, β-unsaturated ketones [50]. A change in the intensity ratio of the 2995 and 2945 cm^−1^ bands can be seen, which confirms the participation of methyl groups in the ozone oxidation process. The band at 1750 cm^−1^ also changes in intensity, which may be a consequence of disturbances in the structure of the main chain of PLA during ozone oxidation. The 1560 cm^−1^ band, which together with the 1537 cm^−1^ band forms a wide region, is also attributed to vibrations of the carbonyl group involved in the oxidation process and the formation of hydrogen-bonding [51]. After exposure to ozone, a new broad peak in the range of 3200–3800 cm^−1^ can be observed in the spectra of all PLA/NR samples, which is related to the stretching vibrations of single and hydrogen-bonded –OH groups.

Some authors describe an increase in the band at 1300 cm^−1^ with the duration of ozone exposure and note that this may be due to stretching vibrations of the C-O bond in the polylactide matrix [19,52]. The current study also shows this pattern, with a slight change in intensity. The effect of NR on PLA during ozone aging depends on its content in the thermoplastic matrix, as well as on the nature of interfacial interactions and the thickness of the samples. In the works of Rodriguez et al., the effect of the thickness of NR film samples on the depth of the process was discussed [53]. Thus, during ozone treatment of NR samples, only the surface layers of rubber were subjected to the oxidation process, while in the matrix (0.5–170 μm), parallel reactions of crosslinking and destruction of NR macrochains occurred. The third layer was a non-reactive NR layer where the film thickness was greater than 170 µm. Probably, the ozone diffusion issue is more relevant for films, since fibrous composites have a very developed surface and a small thickness from several tens of nm to 100 μm. In the case of PLA/NR fibers, the thermoplastic to elastomer ratio and the interfacial interactions described earlier are more important [54,55].

**Figure 12:**
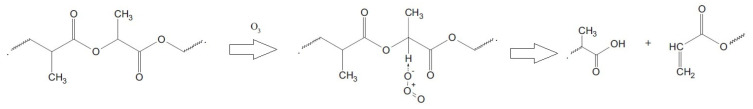
Degradation of PLA during ozone oxidation.

It should be noted that in 100% PLA fiber and in PLA/NR fibers, the appearance of identical bands is noted. This indicates that the disintegration of PLA and PLA/NR fibers under the influence of ozone occurs by the same mechanism, with an attack on the ether group as shown in Figure 12 [19,49].

The addition of NR contributes to the formation of unsaturated compounds with C=C bonds (band at 1537 cm^−1^) and, by affecting the structure and properties of PLA/NR fibers, accelerates the process of ozone aging.

## 4. Conclusions

Nonwoven PLA/NR fibers with NR contents of 5, 10, and 15 wt.% were obtained by electrospinning from solution and subjected to ozone treatment for 800 min at ambient temperature. The effect of ozone on the structure and properties of the resulting PLA/NR fibers was studied by various methods.

The experimental results showed a significant effect of ozone on the morphology, thermophysical and dynamic characteristics, as well as on the chemical structure and rheological properties. The presence of NR in the PLA matrix changes the ratio of amorphous and crystalline phases in PLA/NR samples and enhances the macromolecular dynamics of the system, which is confirmed by a decrease in the correlation time of the probe.

The thermophysical characteristics of PLA/NR fiber samples undergo more pronounced changes compared to 100% PLA. An increase in the degree of crystallinity of PLA/NR fibers by 2–2.5 times and a decrease in T_g_ (by 5–9 °C) and T_cc_, determined by the DSC method, confirm the presence of structural rearrangement in the oxidation process caused by ozone aging. The greatest change in temperature characteristics is noted for the composition with a rubber content of 15 wt.%, where T_g_ and T_cc_ decrease by 9 and 11 °C, respectively. The change in the color of PLA/NR fibers and yellowing observed by optical microscopy, as well as the increase in melt flowability, confirms the occurrence of oxidative degradation in the polymer system.

The FTIR spectra recorded before and after ozone aging indicate that ozone causes decomposition of the polymer matrix, which leads to the appearance of new bands in the spectra related to –OH groups and –C=C– bonds. The totality of the obtained data allows us to conclude that ozone has a significant effect on PLA/NR nonwoven fibers, and the incorporation of natural rubber into the polylactide matrix accelerates the process of ozone oxidation. This study adds to the knowledge about the influence of the second component in the thermoplastic-elastomer system during ozone oxidation. In further research, it is planned to establish the effect of the matrix structure on the kinetics of oxygen absorption and to evaluate the change in properties after oxidation.

## Figures and Tables

**Table 1:** Thermophysical characteristics of the reference nonwoven PLA/NR fibers and after ozone aging.

NR, wt.%	Tg, °C (Δ ± 0.3 °C)			Tcc, °C (Δ ± 0.3 °C)			Tm, °C (Δ ± 0.3 °C)			χ_c_, % (Δ ± 1%)		
	0 min	400 min	800 min	0	400	800	0	400	800	0	400	800
0	66	64	61	101	102	103	167	167	167	16	17	20
5	67	64	61	104	102	100	168	167	167	11	15	23
10	68	62	59	104	101	98	168	167	167	13	18	25
15	68	63	59	105	100	94	168	167	167	12	20	30

**Table 2:** Rotational correlation time of the probe (τ_c_ × 10^−10^ s^−1^) of PLA/NR fibers at different ozone exposure times.

NR, wt.%	Rotational Correlation Time, τ_c_ × 10^−10^, s^−1^			
	0 min	200 min	400 min	800 min
0	80.2 ± 0.3	65.0 ± 0.5	46.6 ± 0.7	34.3 ± 0.5
5	42.4 ± 0.6	34.1 ± 0.7	28.7 ± 0.5	14.1 ± 0.6
10	31.3 ± 0.3	22.0 ± 0.4	15.5 ± 0.6	8.2 ± 0.4
15	20.5 ± 0.4	13.5 ± 0.5	10.2 ± 0.4	4.5 ± 0.2

The table shows average values.

**Table 3:** The average diameter (*d_av_*) and bulk density of PLA/NR fibers before and after ozone treatment for 800 min.

NR Content, wt.%	*d_av_*, µm	Bulk Density g/cm^3^
0	5.7–7.4/5.6–7.3	0.160/0.157
5	6.4–8.5/6.2–8.4	0.172/0.167
10	6.7–9.3/6.4–9.2	0.178/0.172
15	6.7–9.9/6.3–9.7	0.182/0.174

## Data Availability

The original contributions presented in this study are included in the article/Supplementary Materials. Further inquiries can be directed to the corresponding author.

## Supplementary Materials

The following supporting information can be downloaded at: https://www.mdpi.com/article/10.3390/polym17152102/s1, Figure S1: FTIR-ATR spectra of PLA/NR fibers with different NR content, wt.%: 0 (a), 5 (b), 10 (c), 15 (d): black—initial, red—800 min of ozone aging.

## Abbreviations

The following abbreviations are used in this manuscript:

PLA	Polylactide
NR	Natural Rubber
DSC	Differential scanning calorimetry
FTIR	Fourier Transform Infrared Radiation
MFR	Melt Flow Rate
XRD	X-ray Diffraction
ATR	Attenuated total reflection

## References

[1] Kim J., Kang S., Seong I., Jeon J.W., Lee D., Kim J.-H., Kwon D.-J. (2025). Advancing CFRP durability: Interfacial and weathering performance of epoxy and acrylic matrices. Compos. B Eng..

[2] Podzorova M., Tertyshnaya Y., Khramkova A., Ivanitskikh A. (2022). Effect of UV-Irradiation and Ozone Exposure on Thermal and Mechanical Properties of PLA/LDPE. Films Modern Trends in Manufacturing Technologies and Equipment. Mater. Res. Proc..

[3] Zhao W., He J., Yu P., Jiang X., Zhang L. (2023). Recent progress in the rubber antioxidants: A review. Polym. Degrad. Stab..

[4] Rose K., Tenberge K.B., Steinbüchel A. (2005). Identification and characterization of genes from *Streptomyces* sp. strain K30 responsible for clear zone formation on natural rubber latex and poly(cis-1,4-isoprene) rubber degradation. Biomacromolecules.

[5] Tertyshnaya Y.V., Podzorova M.V., Khramkova A.V., Ovchinnikov V.A., Krivandin A.V. (2023). Structural Rearrangements of Polylactide/Natural Rubber Composites during Hydro- and Biotic Degradation. Polymers.

[6] Podzorova M.V., Tertyshnaya Y.V. (2021). Kinetic patterns for thermal oxidation of binary and ternary blends based on polylactide and polyethylene. Russ. Chem. Bull..

[7] Cataldo F. (2001). On the Ozone Protection of Polymers Having Non-Conjugated Unsaturation. Polym. Degrad. Stab..

[8] Kruza M., Lewis A.C., Morrison G.C., Carslaw N. (2017). Impact of Surface Ozone Interactions on Indoor Air Chemistry: A Modeling Study. Indoor Air.

[9] Eng A.H., Kodama S., Nagata K., Kawasaki H. (1998). Reaction of Moist Ozone with Natural Rubber. J. Rubb. Res..

[10] Treib C., Loos K., Johlitz M., Lion A. (2022). Ozone ageing: Experimental methods on pristine and protected natural rubber. Contin. Mech. Thermodyn..

[11] Cataldo F. (2019). Protection Mechanism of Rubbers from Ozone Attack. Ozone Sci. Eng. J. Int. Ozone Assoc..

[12] Zhou H., Liu J., Xia H., Zhang Q., Ying T., Hu T. (2015). Removal and reduction of selected organic micro-pollutants in effluent sewage by the ozone-based oxidation processes. Chem. Eng. J..

[13] Huang C., Shi Y., El-Din M.G., Liu Y. (2016). Optimization of ozonation combined with integrated fixed-film activated sludge (IFAS) in the treatment of oil sands process-affected water (OSPW). Int. Biodeterior. Biodegr..

[14] Wang L., Luo Y., Luo X., Wang R., Li Y., Shao H., Chen Z. (2016). Effect of deoxynivalenol detoxification by ozone treatment in wheat grains. Food Control.

[15] Zeng Z., Zou H., Li X., Arowo M., Sun B., Chen J., Chu G., Shao L. (2013). Degradation of phenol by ozone in the presence of Fenton reagent in a rotating packed bed. Chem. Eng. J..

[16] Kuleznev V.N., Shershnev V.A. (2014). Chemistry and Physics of Polymers.

[17] Kamaruddin S., Muhr A.H. (2018). Investigation of Ozone Cracking on Natural Rubber. J. Rubber Res..

[18] Zheng T., Zheng X., Zhan S., Zhou J., Liao S. (2021). Study on the ozone aging mechanism of Natural Rubber. Polym. Degrad. Stab..

[19] Olewnik-Kruszkowska E., Nowaczyk J., Kadac K. (2016). Effect of ozone exposure on thermal and structural properties of polylactide based composites. Polym. Test..

[20] Olewnik-Kruszkowska E., Nowaczyk J., Kadac K. (2017). Effect of compatibilizig agent on the properties of polylactide and polylactide based composite during ozone exposure. Polym. Test..

[21] Abdullah M.F., Andriyan A., Muhamad F., Ang B.C. (2021). Effect of core-to-shell flowrate ratio on morphology, crystallinity, mechanical properties and wettability of poly(lactic acid) fibers prepared via modified coaxial electrospinning. Polymer.

[22] Karpova S.G., Tertyshnaya Y.V., Podzorova M.V., Popov A.A. (2021). Effect of Exposure in Aqueous Medium at Elevated Temperature on the Structure of Nonwoven Materials Based on Polylactide and Natural Rubber. Polym. Sci. Ser. A.

[23] Lv S., Zhang Y., Tan H. (2019). Thermal and thermo-oxidative degradation kinetics and characteristics of poly (lactic acid) and its composites. Waste Manag..

[24] Dana H.R., Ebrahimi F. (2023). Synthesis, properties, and applications of polylactic acid-based polymers. Polym. Engin. Sci..

[25] Tertyshnaya Y.V., Lobanov A.V., Morokov E.S., Buzanov G.A., Abushakhmanova Z.R. (2023). Polylactide—Meso-Substituted Arylporphyrin Composites: Structure, Properties and Antibacterial Activity. Polymers.

[26] Shekhar N., Mondal A. (2024). Synthesis, properties, environmental degradation, processing, and applications of Polylactic Acid (PLA): An overview. Polym. Bull..

[27] Steinbüchel L.A. (2005). Biodegradation of Natural and Synthetic Rubbers.

[28] Povernov P.A., Shibryaeva L.S., Lyusova L.R., Kotova S.V., Zykova A.K. (2022). The Influence of Mixing Conditions on the Morphology of Poly-3-Hydroxybutyrate and Nitrile-Butadiene Rubber Polymer Compositions. Polymer Sci. Ser. D.

[29] Tertyshnaya Y.V., Podzorova M.V., Karpova S.G., Krivandin A.V. (2024). Structural Features of Polylactide and Natural Rubber Films Produced by Solution Casting. J. Phys. Chem. B.

[30] Xu C., Yuan D., Fu L., Chen Y. (2014). Physical blend of PLA/NR with co-continuous phase structure: Preparation, rheology property, mechanical properties and morphology. Polym. Test..

[31] Lim L.-T., Auras R., Rubino M. (2008). Processing technologies for poly(lactic acid). Prog. Polym. Sci..

[32] Pavićević A., Luo J., Popović-Bijelić A., Mojović M. (2017). Maleimido-proxyl as an EPR spin label for the evaluation of conformational changes of albumin. Eur. Biophys. J..

[33] (1995). Textiles—Test Methods for Nonwovens.

[34] (2021). Plastics—Determination of the Melt Mass-Flow Rate (MFR) and Melt Volume-Flow Rate (MVR) of Thermoplastics—Part 1: Standard Method.

[35] Kuleznev V.N. (1980). Polymer Blends.

[36] Di Vona M.L., Drioli E., Giorno L. (2014). Annealing of Polymer Membranes. Encyclopedia of Membranes.

[37] Jayanth N., Jaswanthraj K., Sandeep S., Mallaya N.H., Siddharth S.R. (2021). Effect of heat treatment on mechanical properties of 3D printed PLA. J. Mech. Behav. Biomed. Mater..

[38] Hart K.R., Dunn R.M., Wetzel E.D. (2020). Increased fracture toughness of additively manufactured semi-crystalline thermoplastics via thermal annealing. Polymer.

[39] Gao P., Alanazi S., Masato D. (2024). Crystallization of Polylactic Acid with Organic Nucleating Agents under Quiescent Conditions. Polymers.

[40] Zhang J., Tashiro K., Tsuji H., Domb A.J. (2008). Disorder-to-order phase transition and multiple melting behavior of poly(l-lactide) investigated by simultaneous measurements of WAXD and DSC. Macromolecules.

[41] Kim J., Kim Y., Cho Y. (2025). Estimation of Synthetic Rubber Lifespan Based on Ozone Accelerated Aging Tests. Polymers.

[42] Podzorova M.V., Tertyshnaya Y.V. (2024). Dynamics of the degradation of polylactide-natural rubber films under the influence of UV-irradiation. J. Phys. Chem. B.

[43] Ol’khov A.A., Kucherenko E.L., Zhul’kina A.L., Iordanskii A.L., Shibryaeva L.S., Tertyshnaya Y.V., Kovaleva A.N. (2017). Resistance to thermal oxidation of ethylene propylene rubber and polyhydroxybutyrate blends. Int. Polym. Sci. Technol..

[44] Margolin A.L., Vorontsov N.V., Monakhova T.V., Popov A.A. (2024). Thermal oxidation of blends of polypropylene and polyamide 6/66. Effect of inhibition. Polym. Degrad. Stab..

[45] Vorontsov N.V., Popov A.A., Margolin A.L. (2019). The effect of thermal oxidation on biodegradation of isotactic polypropylene, polyamide 6/66-4 and their mixtures. AIP Conf. Proc..

[46] Tertyshnaya Y.V., Skorokhodova A.N., Anpilova A.Y., Olkhov A.A. (2024). Promotive Effect of Non-Woven Polylactide/Natural Rubber Composites on Growth and Biochemical Constituents of Purple Basil (*Ocimum basilicum* L.). J. Compos. Sci..

[47] Molinaro S., Romero M.C., Boaro M., Sensidoni A., Lagazio C., Morris M., Kerry J. (2013). Effect of nanoclay-type and PLA optical purity on the characteristics of PLA-based nanocomposite films. J. Food Eng..

[48] Wang X., Pan H., Yang K., Zhang P. (2022). Cracking, structural, and mechanical property changes of SIBR and related elastomers during the ozone aging process. Polym. Degrad. Stab..

[49] Gupta A., Kumar N., Sachdeva A. (2024). Factors affecting the ageing of polymer composite: A state of art. Polym. Degrad. Stab..

[50] Zaidi L., Kaci M., Bruzaud S., Bourmaud A., Grohens Y. (2010). Effect of natural weather on the structure and properties of polylactide/Cloisite 30B nanocomposites. Polym. Degrad. Stab..

[51] West C., McTaggart R., Letcher T., Raynie D., Roy R. (2019). Effects of gamma irradiation upon the mechanical and chemical properties of 3D-printed samples of polylactic acid. J. Manuf. Sci. Eng..

[52] Krikorian V., Pochan D.J. (2005). Crystallization behavior of poly(L-lactic acid) nanocomposites: Nucleation and growth probed by infrared spectroscopy. Macromolecules.

[53] Rodrigues F.H.A., Santos E.F., Feitosa J.P.A., Ricardo N.M.P.S., Heatley F. (2004). Ozonation of unstretched natural rubber film from *Hevea brasiliensis* studied by ozone consumption and ^13^C NMR. Polym. Int..

[54] Tertyshnaya Y., Karpova S., Moskovskiy M., Dorokhov A. (2021). Electrospun Polylactide/Natural Rubber Fibers: Effect Natural Rubber Content on Fiber Morphology and Properties. Polymers.

[55] Bitinis N., Verdejo R., Maya E.M., Espuche E., Cassagnau P., Lopez-Manchado M.A. (2012). Physicochemical properties of organoclay filled polylactic acid/natural rubber blend bionanocomposites. Compos. Sci. Technol..

