# Supplementary material for: Effect of Ozone on Nonwoven Polylactide/Natural Rubber Fibers
Source: Polymers (Basel). 2025 Jul 31;17(15):2102. doi: 10.3390/polym17152102 (PMC12349087; doi:10.3390/polym17152102)
Supplement: Supplementary file 1 [file polymers-17-02102-s001.zip › polymers-3782758-supplementary.pdf]

SUPPLEMENTARY FILE (Yulia Tertyshnaya *et al.*)

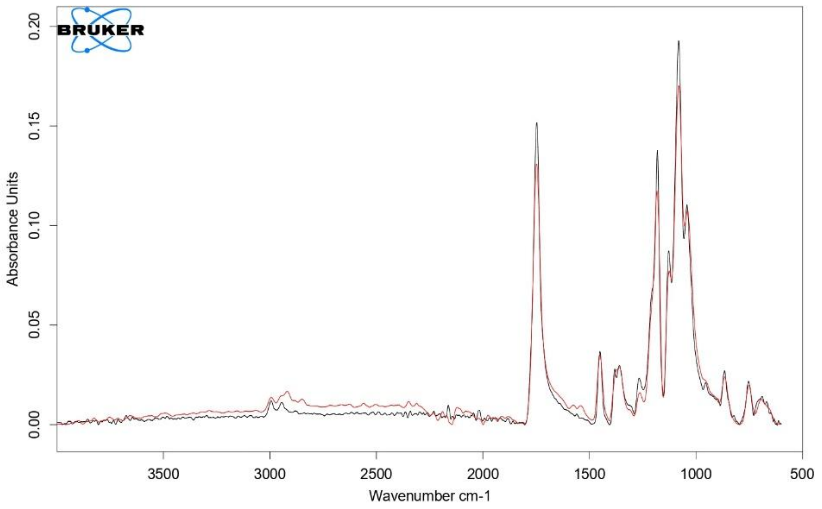

a

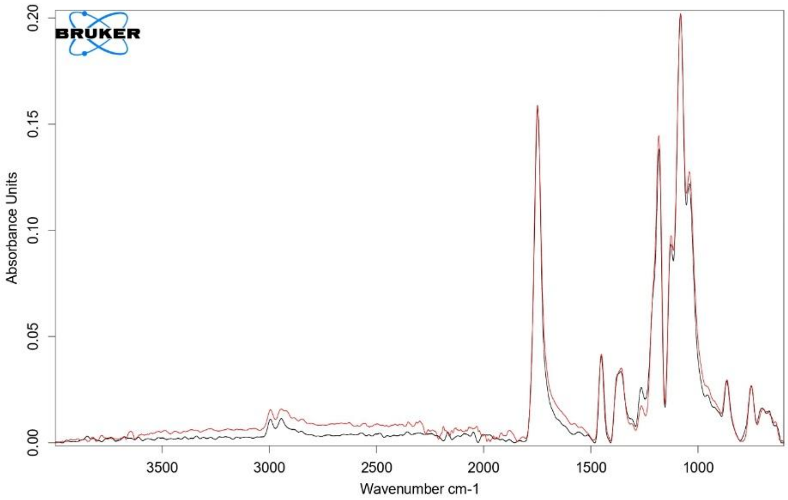

b

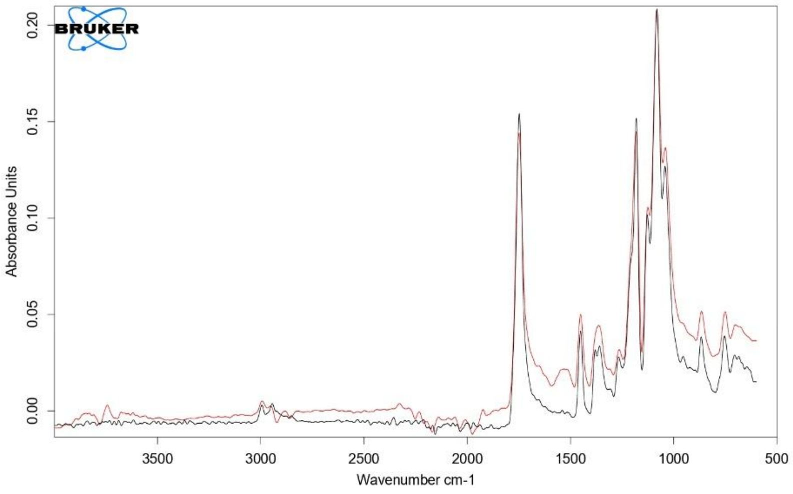

c

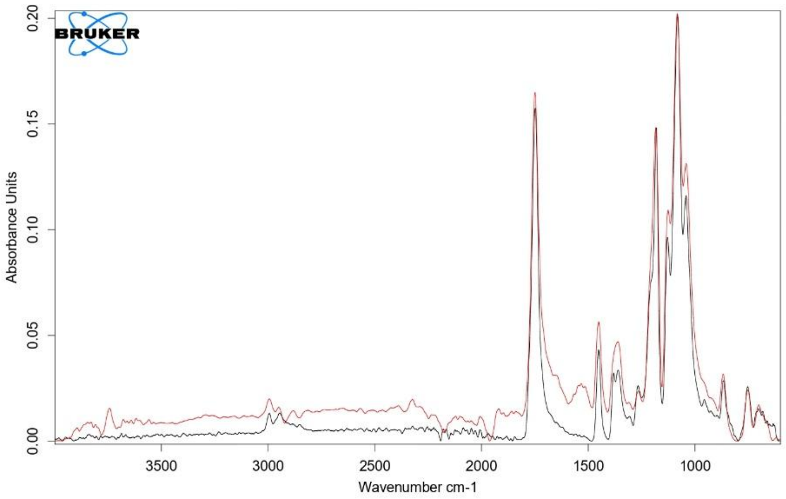

d

Figure S1. FTIR-ATR spectra of PLA/NR fibers with different NR content, wt.%: 0 (a), 5 (b), 10 (c), 15 (d): black – initial, red – 800 min of ozone aging.
